# Ultrafast Laser Writing Deep inside Silicon with THz-Repetition-Rate Trains of Pulses

**DOI:** 10.34133/2020/8149764

**Published:** 2020-05-14

**Authors:** Andong Wang, Amlan Das, David Grojo

**Affiliations:** Aix-Marseille Univ., CNRS, LP3 UMR 7341, 13009 Marseille, France

## Abstract

Three-dimensional laser writing inside silicon remains today inaccessible with the shortest infrared light pulses unless complex schemes are used to circumvent screening propagation nonlinearities. Here, we explore a new approach irradiating silicon with trains of femtosecond laser pulses at repetition rates up to 5.6 THz that is order of magnitude higher than any source used for laser processing so far. This extremely high repetition rate is faster than laser energy dissipation from microvolume inside silicon, thus enabling unique capabilities for pulse-to-pulse accumulation of free carriers generated by nonlinear ionization, as well as progressive thermal bandgap closure before any diffusion process comes into play. By space-resolved measurements of energy delivery inside silicon, we evidence changes in the interplay between detrimental nonlinearities and accumulation-based effects. This leads to a net increase on the level of space-time energy localization. The improvement is also supported by experiments demonstrating high performance for 3D laser writing inside silicon. In comparison to repeated single pulses, irradiation with trains of only four-picosecond pulses with the same total energy leads to an apparent decrease of the energy threshold for modification and drastic improvements on the repeatability, uniformity, and symmetricity of the produced features. The unique benefits of THz bursts can provide a new route to meet the challenge of 3D inscription inside narrow bandgap materials.

## 1. Introduction

Infrared ultrashort laser pulses open a unique possibility for tailoring material properties with three-dimension (3D) control, enabling applications in various fields [1] such as optical communications [2], quantum photonics [3, 4], microfluidics, and lab-on-chip applications [5, 6]. Despite its successful applications in wide-bandgap dielectrics, achieving permanent modifications inside narrow bandgap materials remains very challenging due to higher refractive index, easier electron excitation, and lower self-focusing threshold that create unfavorable conditions for high space-time energy localization [7]. Several methodologies have been recently proposed to confine enough energy to exceed the thresholds for permanent changes in bulk silicon (Si). On this prospect, long pulses (nanosecond duration or more) are clearly more favorable because of reduced peak power and a thermal runaway beneficial for energy deposition. However, the level of controllability on the morphology, type, and resolution of the modifications is very limited due to the thermal nature of material transformations [8–12]. For increased control, the ultrafast optical breakdown regimes are thus attractive. However, experiments show that these regimes are hardly accessible in Si due to severe self-screening and nonlinear delocalization effects [13–15].

During the last few years, several strategies have been proposed to solve this problem. Among them, the use of solid immersion focusing schemes has revealed that hyperfocusing allows circumventing the limitations and achieving modifications in Si with single femtosecond pulses [7]. However, the contact nature of the required arrangement creates severe difficulties for practical applications. Looking for optimizations in the time domain, several works report successful bulk Si modification by using high repetition rate femtosecond laser pulses. In particular, direct waveguide writing is achieved using 350 fs at 250 kHz [16] by Pavlov et al. and using 800 fs pulses at 400 kHz [17] by Matthäus et al. In these reports, thermal and free-carrier pulse-to-pulse transient accumulations are hypotheses to explain the modifications at high repetition rate. However, this remains contradictory with the characteristic nanosecond timescale for heat dissipation and the decay of microplasmas observed in Si [18].

To rely on accumulation processes in Si, much higher repetition rates are in principle required. For this reason, we experimentally investigate in this work the bulk Si response to trains of pulses at repetition rates up to THz level (hereafter called *bursts*). We introduce in this way new control parameters for multitimescale optimizations. The method is loosely analog to recent works demonstrating enhanced surface ablation efficiencies with femtosecond laser sources in burst modes [19]. The latter rely on GHz laser sources delivering trains of tens to hundreds of pulses. The GHz repetition rate is particularly appropriate for surface ablation because it allows benefiting from both the so-called ablation cooling effect and heat-accumulation-based effects lowering the ablation thresholds [19–21]. Obviously, ablation cooling relying on progressive evacuation of hot materials is not accessible for subsurface applications. However, one can still expect that the other major benefits of burst mode irradiations remain applicable. After making this comment, it is natural to turn directly toward repetition rates even higher than GHz so that accumulation processes can give their full potential before energy dissipation processes come into play in Si.

In this paper, we use a stack of birefringent crystals to readily generate and apply trains with a maximum of 64 pulses at 1550 nm wavelength. In addition to drastically simplifying the required laser technology to achieve burst mode of irradiation, the method gives the possibility to access repetition rates exceeding 5 THz. With such trains, we show how a timescale analysis of the problem allows multitimescale optimizations for energy confinement. By space-resolved measurements of energy delivery inside Si, we evidence that the modified interplay between detrimental propagation nonlinearities and energy deposition accounting for the accumulation-based effects leads to a net increase on the level of space-time energy localization. The benefits of THz burst mode of irradiation are then exemplified by a series of experiments demonstrating reduced energy thresholds and improved controllability for bulk inscription inside Si.

## 2. Results

### 2.1. Timescale Analysis and Burst Generation

**Figure 1:**
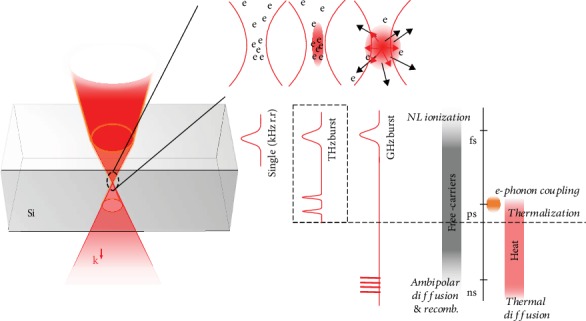
Timescales and schematic representation of expected accumulation effects in Si. Strong single pulse will develop plasma in prefocal region that limits the intensity and energy deposition near the focus. With a THz-repetition-rate train of weaker pulses, one can accumulate carrier at the focus. With train duration exceeding electron-phonon coupling time but below diffusion times, we can expect local heating gradually increasing absorption during the train. At repetition rates down to GHz, thermal and electronic diffusions come into play that may limit energy localization.

By applying trains of ultrashort pulses, we introduce new possibilities to control the interaction dynamics by changing the pulse-to-pulse separation, the number of pulses, and consequently the duration of the interactions. As sketched in Figure 1, comparing to repeated single pulses (at kHz repetition rate for example), energy deposition with bursts at high repetition rates can benefit from accumulating free carriers and temperature near the focus for two main reasons. On the one hand, for a given absorbed energy density, we can expect to maintain the intensity and peak power of the pulses at low level and avoid partially the strong nonlinear propagation effects delocalizing the laser energy prior to the focal region. Depending on situations, these include highly efficient multiphoton absorption that progressively depletes the pulse energy before reaching the focal region, Kerr effect-based distortions due to the low critical power in Si and strong plasma shielding [7, 22]. On the other hand, the burst can benefit from local bandgap closure with progressive temperature rise during the train. For this aspect, one can refer to Varshni's empirical expression [23] which leads to an estimation of a bandgap decrease below 0.8 eV for a temperature of 1100 K. This corresponds to linear absorption of 1550 nm radiation below the melting threshold of Si (*T*_melt_ = 1687 K), but we can also expect an increased two-photon absorption at even lower temperature. This, combined with inverse Bremsstrahlung absorption assisting energy deposition as soon as nonlinear ionization is initiated, makes that an effective reduction of modification thresholds can be locally expected with burst irradiation.

However, it is important to highlight that accumulation benefits are obviously conditioned by a separation between adjacent pulses smaller than the timescales of all electronic and thermal dissipation processes. To justify our vision and confirm that the characteristics of pulse trains at THz repetition rates are appropriate, we must look at the timescales of all electronic and thermal processes. Those are sketched in Figure 1. Under similar conditions, we have measured a recombination time and ambipolar diffusion coefficient leading to a microplasma lifetime of about 2.5 ns in Si [18]. By a simple estimate of the thermal diffusion time *t* = *l*^2^/*D* associated to a length *l* = 1 *μ*m (the typical dimension of focus), the thermal diffusivity coefficient for Si at room temperature *D* = 0.86 cm^2^/s leads to 10 ns as the characteristic time for the thermal energy to diffuse away from the interaction region. Then, we can conclude that pulse trains exhibiting repetition rates higher than GHz must allow efficient local thermal and free-carrier accumulation. It is also interesting to note at this stage the relatively similar characteristic time for carrier transport and thermal diffusion. This may make it difficult to decouple these two contributions with fine tuning between THz and GHz repetition rates.

**Figure 2:**
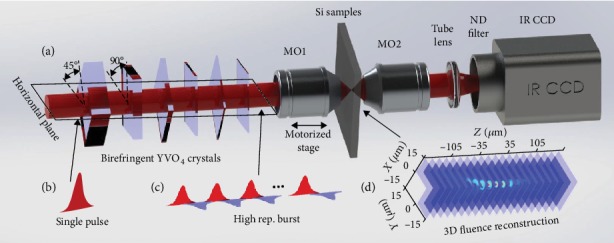
Illustrations of main experimental processes. (a) Experimental setup for the generation of trains of ultrashort pulses and reconstruction 3D fluence distributions inside Si. (b, c) Temporal pulse shapes and associated polarization orientations before and after the crystals. (d) 3D laser fluence mapping in the focal region reconstructed from a succession of captured images applying a z-scan procedure along the laser propagation axis.

To satisfy this requirement of high repetition rate, we rely on an engineered arrangement of birefringent crystals [24, 25] to split a pulse in delayed replica. This external laser cavity method is sketched in Figure 2. It allows in-line collinear splitting and avoids the need for building interferometers as well as tedious alignment procedures for splitting and recombining beams as many as pulses in the intended trains [26, 27]. The delay between pulses can be precisely controlled by adjusting the thicknesses of the birefringent crystals. The number of replicas can be readily increased by adding crystals of appropriate thicknesses. In this paper, trains of 2, 4, 8, 16, 32, and 64 pulses are produced with 6 crystals of different predefined thicknesses (detailed characteristics of the trains and associated splitting methods can be found in Supplementary Note 1). For the 64-pulse train, the shortest interpulse delay of 180 fs between pulses corresponds to an irradiation at a repetition rate of ≅5.6 THz. The obtained trains of pulses for all crystal combinations are systematically verified by using a long-scan autocorrelator (see Supplementary Note 2). It is worth noting here that the splitting methodology leads to a succession of pulses with alternating orthogonal polarizations (as shown in Figure 2(c)) so that no interference is expected even in case of partial temporal overlap between adjacent pulses.

### 2.2. Ultrafast Accumulation of Absorbed Energy Density

It is important to highlight that the burst strategy holds only if one can develop a highly contrasted interaction near focus because all the accumulation effects become detrimental if they develop preferentially in the prefocal region. This question relies on the complex interplay in time and space between energy deposition and propagation effects that can only be rigorously accessed by 3D modeling [28, 29]. The latter is already a complex subject for ultrafast single-pulse interactions and it would become even more so for pulse trains raising fundamental questions that are out of scope for this work. For a model-independent study of the bulk-Si response to burst irradiations, we have performed an experiment which aims at mapping in 3D space the absorbed energy density. This is based on careful numerical analysis of measured fluence distributions. As shown in Figure 2(d), an infrared imaging system is built behind the irradiated sample. A microscope objective lens (MO1) of 0.45 numerical aperture (NA) is used to focus the pulse trains and a second microscope objective (MO2) of higher numerical objective (NA = 0.7, ×100) collects all the transmitted light to re-image the fluence distribution in the plane of an InGaAs array without low pass spatial filtering even in the case of diffracted or defocused beams due to the interaction in Si. Then, by applying a z-scan procedure across the back surface of the sample with the conditions described in Section 4 (Materials and Methods, see 4.2), we can retrieve the 3D fluence distributions delivered near focus as if it were in the bulk (details can be found also in Supplementary Note 1).

**Figure 3:**
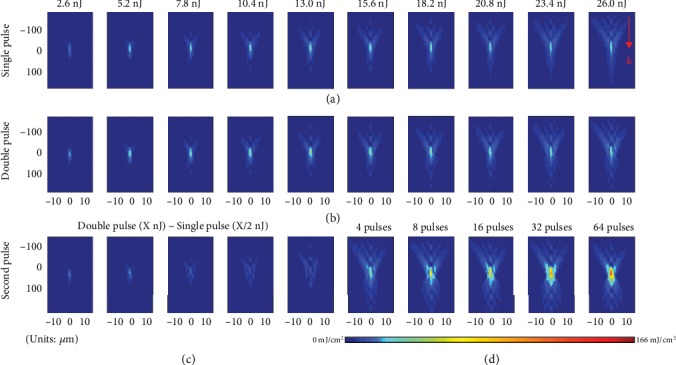
Comparison of fluence distributions (cross-sections across optical axis) achieved near focus. Fluence distributions for (a) single-pulse and (b) double-pulse irradiations at different incoming energy. (c) Fluence contributions of the second pulse in the double-pulse irradiation. (d) Fluence distributions for trains with different number of pulses at constant total energy of 26 nJ.

**Figure 4:**
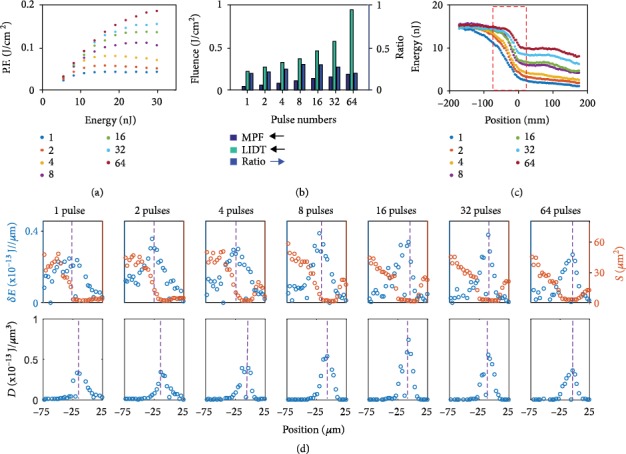
Analyses of the energy densities achieved in Si with different number of pulses in the trains. Different number of pulses from 1 to 64 in the applied THz burst: (a) delivered peak fluence (P.F.) inside Si as a function of burst energy; (b) maximum peak delivered fluence (MPF), laser-induced damage threshold (LIDT), and ratio between them; (c) total burst energies as a function of propagation distance inside Si (zero position representing the geometrical focusing plane). The red box part is further analyzed in (d) showing the pulse energy reduction per distance unit (*δE*, blue) and the apparent beam size (*S*, half maximum area, red curve) in the first row and corresponding absorbed energy densities (*D*) at different positions in the second row.

As a reference, the delivered fluence distributions for single pulses focused with different energies are shown in Figure 3(a). To quantitatively compare the delivered fluences to burst cases, we plot the peak delivered fluence from measured distributions as function of incident pulse energy in Figure 4(a). As the pulse energy grows up to 10 nJ, the maximum fluence increases but a saturation is systematically observed for higher incoming pulse energies. This observation is very similar to what we recently reported at 1300 nm for various focusing conditions [7]. Repeating the measurements with bursts of 2, 4, 8, 16, 32, and 64 subpulses, it is interesting to note on Figure 4(a) an increasing fluence clamping level and a saturation occurrence at higher incoming energy with the increase of the number of pulses in the burst. The fluence clamping phenomenon was previously demonstrated as a consequence to the strong nonlinear absorption and plasma shielding effects developed in the prefocal region, preventing most of the pulses to reach the geometrical focus inside Si [7]. This is clearly evidenced in the captured fluence maps given in Figure 3(a) exhibiting a strong dissymmetry between the pre- and post-focal regions developed as the single pulse energy is increased. The much weaker intensity found after the geometrical focus evidences that the last sources of losses are near the focus where we expect the highest peak intensity, but the clamping shows the importance of other losses in the prefocal region. A first conclusion with these burst measurements here is that similar strong prefocal interaction is also clearly developing but less efficiently as shown with the increased saturation levels (Figure 4(a)).

For assessment of the individual contribution of each pulse in the burst, Figure 3(b) makes a direct comparison with the fluence distribution obtained with the double-pulse case. A first observation is an increase of the peak fluence in comparison to the single-pulse situation at the same energy. This can be directly attributed to a decrease of the intensity and associated self-screening nonlinear effects due to energy sharing between the two pulses. To access the contribution of each pulse in the double-pulse sequences to the fluence maps, we subtract in Figure 3(c) the intensity map of the first pulse (single-pulse case at half energy). By this way, we obtain the fluence contribution of the second pulse for all double-pulse cases. Comparing the contribution of the first pulse, the second pulse indeed adds energy to the foci. However, at high pulse energy, the second pulse delivers much less energy than the lower energy case, demonstrating that there is a maximum incoming energy that should not be exceeded for an efficient accumulation strategy. This is important to avoid shielding effects similar to those of the single-pulse case and is a vision consistent with Figure 4(a) where we find only a modest increase of the clamping level of the peak delivered fluence with double-pulse irradiations in comparison to single pulses.

To fully evaluate the range of optimization by this approach, we have increased progressively the number of pulses in the applied trains. The comparisons are made at constant total burst energy so that the energy of the individual pulses in the trains is constantly decreased as we increase the number of pulses. By making an analysis similar to the one used for the double-pulse case, we can compare directly the fluence delivered by the first and second half of the pulse trains. We found that the second half of the burst can still reach the foci plane with high energy (Supplementary Note 3), proving that higher number of pulses can benefit the fluence delivery to the foci. In Figure 3(d), we show the intensity distribution at the same energy of 26 nJ but different number of pulses in the applied trains (the intensity maps at other energies are shown in Figure S5, Supplementary Note 3). The peak fluence shown in Figure 4(a) indicates that the saturation energy increases when the number of pulses increases. The maximum fluence has been increased by a factor exceeding 4 for 64 pulses in comparison to single pulse. It clearly shows that the delivered energy density at the focusing plane increases as the number of subpulses increases in the burst, but it also shows that the distributions progressively resymmetrize by increasing the number of pulses. The latter observation evidences that nonlinear absorption vanishes due to lower peak intensities in the pulse trains.

While this spatially resolved diagnostic allows monitoring the local delivered fluence, it is worth noting that it does not allow evaluating how far from modification thresholds are the conditions applied to the matter. This is because the thresholds are expected to change with the temporal characteristics of the trains. To solve this question, one would ideally need to retrieve the absorbed energy density distributions but only the delivered fluence is directly accessible in the experiments. Rigorous mapping of the absorbed energy densities in such 3D nonlinear propagation problem requires in principle to turn to comparison with simulations [12, 28]. However, to avoid the complexities mentioned above, we decided to rely on more simple model-independent estimations.

First, we complement our measurements with surface damage threshold measurements for all applied bursts (Supplementary Note 4). In Figure 4(b), we compare the maximum peak delivered fluences (MPF) inside Si for the pulse trains with that of the fluence threshold for laser-induced damage threshold (LIDT) on the surface. Assuming that the fluence threshold for bulk modification is a target following a trend similar to the measured surface threshold, it is informative to note that the LIDT grows more rapidly than the MPF with the number of pulses in the applied trains. Interestingly, when we plot directly the ratio between MPF and LIDT for surfaces, as it is shown in Figure 4(b), we note that it first increases and then decreases after 16 pulses. This implies that there is likely an optimum number of pulses to apply conditions to matter that are the most inclined to lead to permanent modifications.

The general conclusion on the existence of an optimal number of pulses must hold because it would otherwise lead to the absurd conclusion that a CW laser is the most appropriate regime. However, it is also important to mention at this stage that it remains difficult to evaluate the reliability of this estimation on the optimal number of pulses derived by these measurements (Figure 4) because of important assumptions that are not rigorously fulfilled like (i) a fluence threshold for modification identical for bulk and surface and (ii) the absence of the temporal reshaping before target in the bulk for appropriate comparison with surface experiments (no interaction in air at considered intensities).

There are usually two general criteria to predict the threshold for femtosecond laser-induced modifications. The first one is the free-carrier density reaching the critical plasma density [30]. The second one is more simply based on a threshold on the absorbed energy density as, for instance, the energy density for Si melting of ≅5 kJ/cm^3^. Confronting the available fluence distribution data to these simple energetic considerations leads interestingly to a conclusion very similar to the one above consisting in comparing the delivered peak fluences to the surface damage thresholds. The results are displayed in Figure 4. In Figure 4(c), we show the pulse energy as a function of propagation distance obtained by integration of the z-scan images (26 nJ case). For single pulse, the energy losses occur primarily in a region of about 150 *μ*m prior to focus (zero position is the focal plane). Before and after this range, the energy constantly reduces but at very low rate evidencing where most of the energy deposition likely occurs. With the different trains, it is interesting to note that the magnitude and distance of significant absorption progressively reduces when increasing the number of pulses. It is also confirmed that the absorption zone is more localized to the geometrical focus for bursts. To quantify the absorbed energy densities, we analyzed these energy reduction rates *δE* (per propagation distance unit) and also the beam sizes (*S*) at different position from -75 *μ*m to 25 *μ*m that are also accessible from the same fluence distribution measurements (26 nJ case). These are shown in the first row of Figure 4(d). The absorption peaks are marked with dashed lines. For the single pulse, the absorption peak is ≅30 *μ*m prior to the focal plane. For a larger number of pulses, the absorption peak gets closer to the focal plane, where the beam size is smaller. The absorbed energy densities (*D*) are calculated based on these data, as shown in the second row of Figure 4(d). This confirms a localization of the energy density. The comparison between pulse trains reveals also a peak absorbed energy density that first gradually increases with the number of pulses up to 16 and then decreases for more pulses.

Similar to the previous analysis based on the MPF-LIDT ratio, the estimates of absorption density show how the THz repetition rate burst mode provides a new degree of optimization to cross the bulk modification threshold that is extremely challenging when single isolated femtosecond pulses are used [7]. While the optimum number of pulses derived from the two analyses is not rigorously identical, we found it is likely between 8 and 16 that is in the range of pulse trains that we can access with our engineered crystal arrangements (up to 64).

### 2.3. Laser Modification Experiments inside Bulk Si

With the knowledge on these new optimization parameters for maximum deposited energy density, we have investigated the potential of the THz burst mode of irradiation for bulk modification in Si. First, it is important to mention that the MPF-LIDT ratios found in the experiments at NA = 0.45 with the 200 fs pulses remain far below 1 (see Figure 4). This means conditions well below permanent modification regimes. We have confirmed this conclusion by damage test studies performed with all available trains under these focusing conditions. The absence of modification for all tested conditions was consistent with the measurements.

Previous works have already revealed two main ways to further increase the locally absorbed energy density in Si. The first one is to rely on tighter focusing [7] and the second one relies on the use of longer pulses up to the picosecond regime [12, 31]. To demonstrate that THz bursts give new optimization parameters for Si bulk modifications, we have chosen to compare the Si modification response with trains of 4.7 ps pulses focused with a NA = 0.85 microscope objective. We work under these conditions because this pulse duration and NA combination stands, as we will see later, just below the threshold for permanent modification in Si with repeated single-pulse irradiations (independently of the pulse energy). By this way, we can detect directly, by the occurrence of internal damage, a benefit from burst mode (even if modest). Because of this relatively long pulse duration and the limited delays accessible with our thickest crystals available, we have compared single-pulse irradiation with trains of only 2 and 4 pulses in these experiments. It is informative at this stage to note that the interpulse delay is 2.72 ps leading to significant pulse-to-pulse temporal overlap.

**Figure 5:**
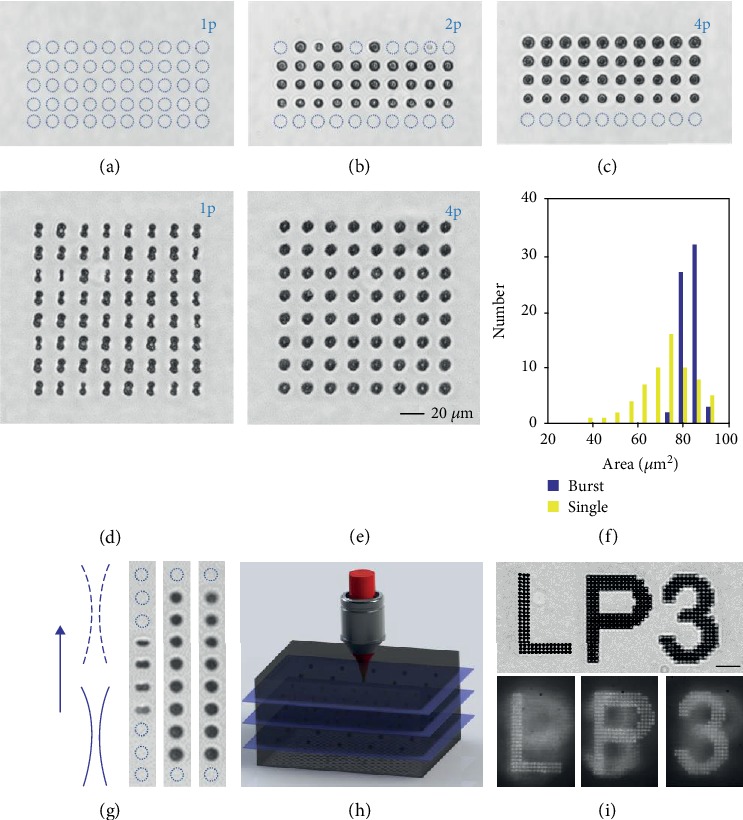
Improved performances for laser writing in bulk Si using ultrafast pulse trains. (a–c) Modification attempts with 4.7 ps (a) single-pulse, (b) double-pulse, and (c) four-pulse irradiations. From top to bottom, the attempts are repeated with identical conditions on each row for different energies: 2.1 *μ*J, 1.9 *μ*J, 1.5 *μ*J, 1.1 *μ*J, and 0.6 *μ*J. The dotted circles represent irradiated sites without apparent modification. (d, e) Modifications obtained with 1.8 *μ*J (d) single-pulse and (e) 4-pulse sequences at the pulse duration of 6.7 ps. (a)–(e) share the same scale bar shown in the corner of (e). (f) Statistics on the apparent area of the modifications (top view) shown in (d) and (e). (g–i) Modifications produced at different depths inside Si. (g) Modification attempts while varying the focusing depth using a z-scan procedure (Δ*z* = 2 *μ*m) centered at 300 *μ*m for which spherical aberration is corrected. The experiment is repeated with 1.5 *μ*J single-pulse (left), double-pulse (center), and four-pulse sequences (right). (h) Illustration of laser writing experiments with 1.9 *μ*J 4-pulse sequences at distinct planes 100 *μ*m, 300 *μ*m, and 500 *μ*m, respectively. Aberrations are corrected for each plane allowing reliable writing of “LP3” with each letter in a separated plane and next to each other (top image) or stacked (bottom images). Scale bar 50 *μ*m.

The results of the experiments comparing by infrared transmission microscopy the permanent modification responses of bulk Si under these conditions are shown in Figure 5. Each irradiation test consists of repeated irradiations with 1000 trains focused at a depth of 0.3 mm below the surface. The irradiation sites are systematically separated by 20 *μ*m. Different energy levels are compared by producing rows of impacts with energies of 2.1 *μ*J, 1.9 *μ*J, 1.5 *μ*J, 1.1 *μ*J, and 0.6 *μ*J, respectively (top to bottom). Each row consists of irradiations with identical conditions repeated 10 times so that we can evaluate not only the modification threshold (when accessible) but also the repeatability of the modifications. The latter is highly informative on the ability to significantly exceed the threshold as previous reports have revealed that near-threshold conditions lead to stochastic behaviors and hardly controllable modifications [12, 31].

An improvement of the conditions for writing is directly evidenced by comparison of Figures 5(a)–5(c). Single-pulse irradiations are systematically below threshold (Figure 5(a)) while all conditions with trains of 4 pulses at energy exceeding 1.1 *μ*J lead to highly reproducible modifications (Figure 5(c)). Interestingly, the sites irradiated with double-pulse trains exhibit smaller features than the 4-pulse trains and low probability for modification occurrence at the maximum tested energy. This is likely due to nonlinear delocalization of the light near critical power for self-focusing [12].

The first important conclusion from this observation for technological perspectives is that the insertion of appropriate crystals can allow making one step up on the deposited energy density and potentially stabilize material writing. The latter has been reported as very sensitive to fluctuations in experiments with femtosecond and picosecond lasers due to clamping at fluences hardly exceeding the modification threshold (when so). For instance, several works report thresholds and morphologies for the modifications which are strongly dependent on the pulse duration [12, 31] and spatial aspects including the beam quality and the focusing depth influencing the profile due to aberration [12, 32]. In addition, some microfabrication demonstrations mention the incapacity to initiate writing from anywhere in 3D space inside Si samples and the requirement to find or create a precursor local change (e.g., focus near surfaces) to seed a continuous 3D writing process [16, 33]. By stepping up the conditions in matter, we believe that pulse splitting can provide a practical solution to this problem.

To illustrate this view, we compare in Figures 5(d) and 5(e) modifications produced with trains of pulses with 6.7 ps duration. This small increase of the pulse duration in comparison to previous cases is made because repeated single pulses meet the condition for possible modification as shown with Figure 5(d) where 64 sites are irradiated with 1000 pulses of 1.8 *μ*J energy (focusing depth = 300 *μ*m). Under these conditions, it is first worth noting a 100%-probability for damage but a rather limited reproducibility as the modifications exhibit all a dissymmetric shape with polarization-dependent orientation but not of identical size. By splitting the same laser energy in 4 pulses, we see in Figure 5(e) that the uniformity of modification is significantly improved. This is quantitatively confirmed with Figure 5(f) showing the distribution of areas of the produced features for the two cases. Larger structures and narrower distributions are produced with the trains of 4 pulses. Interestingly, we also note that the orthogonal flip-flop of polarization between the pulses in the train naturally symmetrizes the modification which may represent also an aspect favorable for many laser writing applications (e.g., waveguide writing). While we concentrate here on the conditions for reliable writing, the mechanisms behind the polarization-dependent morphologies in this report are only partially understood. We will focus future investigations on this particular aspect, but our experiments already reveal that tight in-volume focusing is a requirement to observe this polarization dependence. This does not occur for surface modification with the same laser (see Supplementary Note 5) and/or with the use of lower NA. Interestingly, the polarization-dependent damage response follows a trend very similar to another work [34] by Mori et al. under similar focusing conditions.

Our interpretation is that the better uniformity and repeatability of modification obtained in burst mode relies on the ability (at constant energy) to exceed more significantly the threshold for modification. This must lead to material writing that is more robust to any type of variation affecting the energy delivery to the material. To test this hypothesis, we have repeated the experiments at different focusing depths around 300 *μ*m, the depth for which spherical aberration is corrected with our lens (see experimental section). As shown in Figure 5(g), modifications with single pulses can only be obtained in a range ≅±10 *μ*m around the best focusing condition, while bursts can tolerate a deviation from at least ±20 *μ*m without any noticeable change of the modification observed by IR microscopy (top view). Due to the strong index mismatch at Air-Si surface, strong spherical aberrations are expected to lower the apparent local fluence in the material [32, 35]. In the case of pure linear propagation, simulations based on vectoral theory [36] allow us to predict a drop of the peak fluence of about 6% for a deviation of only 24 *μ*m from the corrected depth (see Supplementary Note 6). While it is difficult to evaluate how this translates in the various nonlinear propagation cases investigated here, it is however a difference that tend to reveal the level of enhancement of the delivered fluence with the burst mode.

To exemplify the improved performance with the train of 4 ps pulses, Figures 5(h) and 5(i) show binary data inscription by dot arrays fabricated at three different depths: 100 *μ*m, 300 *μ*m, and 500 *μ*m.  Figure 5(i) shows three different letters at the different depths but all are corrected for aberrations using the correction collar of the objective. The text is observed with a modest magnification objective (×10, NA = 0.3) focused on the “L” so that the depth of field allows to visualize on the same image the other letters which are deeper in the sample. Interestingly, we note that data can be reliably inscribed in the three planes. Finally, the dark field images at the bottom show the same inscription but with the three letters stacked on top of each other by applying a bottom-up writing procedure. By using a higher NA observation (×20, NA = 0.45), we can clearly distinguish between planes and the best focused image of each plane is shown (a z-scanning observation of the three layers for complete assessment of the situation is also given in Supplementary videos 1 and 2). It is interesting to highlight again the controllability of the produced features in these experiments at very different depths. This remains an aspect that is extremely challenging in some other proposed solutions for advanced laser writing inside Si including hyperfocusing [7], nonlinear feedback with contribution from back-surface reflected beams [8], and sub-MHz repetition rate femtosecond lasers requiring a seeding process to initiate and propagate continuous line writing [16, 33].

## 3. Discussion

In this paper, we have explored an alternative method, based on THz trains of ultrashort pulses to exceed the modification threshold of bulk Si. Bursts with repetition rates up to 5.6 THz and number of pulses up to 64 have been readily generated by a stack of birefringent crystals naturally splitting any incoming ultrashort pulse in a train of delayed replica. The typical picosecond interpulse delay can improve energy deposition due to pulse-to-pulse accumulation before any diffusion process comes into play. Exploiting model-independent strategies to evaluate the applied conditions inside Si with the tightly focused trains of femtosecond pulses, we confirm a benefit for energy deposition in comparison to single-pulse irradiation. Interestingly, the study concludes on a number of pulses in the train that should not be exceeded (in the range 8 to 16) for appropriate excitation build-up and limited retro-reaction delocalizing the beam creating the excitation. To reduce this last limitation, it would be interesting to investigate slightly lower repetition rate. We can actually expect an optimum repetition rate repetition in the range GHz-THz to find the best balance between plasma shielding and heat-assisted absorption as the timescale for electron and heat diffusion are relatively close in Si. This gives a motivation for development of multi-GHz burst mode femtosecond lasers at infrared wavelengths similar to those emerging for surface ablation at shorter wavelengths [20, 21]. The benefits of burst mode of irradiation have been supported by experiments demonstrating improved performance for 3D laser writing inside Si with trains of picosecond pulses.

We have concentrated the investigations on high-resistivity Si. However, the THz repetition rate is so high in this study that our conclusions are not limited to this material. We expect the method can efficiently accumulate energy in any material, including doped-Si for which nonlinear absorption with single pulses does not exhibit dependence to the doping concentration [37]. This is because the interpulse delay is shorter than any characteristic time for energy dissipation from a microscale-processed region. With such high repetition rate, it remains that an important question may arise on the difference on the material response between trains and stretched pulses. While we could not make systematic comparisons to study in details this question, there are already two striking differences in the observations of this report. First is on the symmetry and orientation of the modifications, but this can be directly attributed to the polarization characteristics of the trains in comparison to a single linearly polarized pulse. Second is the absence of modification with THz trains of 64 pulses of 200 fs corresponding to an apparent duration of interaction of about 12 ps while single Gaussian pulses of duration exceeding 7 ps can readily produce permanent modifications. This is a direct evidence that the detailed temporal characteristics of irradiation play a major role in these situations. This gives an additional degree of optimization that should not be overlooked for the development of ultrafast 3D laser writing technologies applicable to narrow gap materials.

## 4. Materials and Methods

### 4.1. Laser Setup

The laser source is a femtosecond laser system (PHAROS, Light Conversion) with a central wavelength of 1028 ± 5 nm and pulse duration ~170 fs. Then, the laser pulses are sent to an optical parameter amplifier (ORPHEUS, Light Conversion) for wavelength conversion. While this offers the possibility to adjust the wavelength, we concentrate here on 1550 nm, the wavelength for maximum conversion efficiency with our system because most studies have been conducted so far in the range 1200-1550 nm corresponding to two-photon absorption initiated interactions in Si. Also, all investigations outside this range have concluded on a relatively modest dependence to the wavelength parameter [22, 38]. The output energy is controlled by a combination of half-waveplate and polarizer. A parallel grating pair is used to change the pulse duration (from 4 ps to 21 ps). The pulse duration is checked by using a home-built long-scan autocorrelator. Details are discussed in supporting information with the whole setup shown in Figure S1.

### 4.2. 3D Fluence Reconstruction

The focusing objectives are from Olympus LCPLN-infrared series, which are designed for semiconductor infrared microscopy. These objectives have adjustable collars for spherical aberration corrections due to silicon. For all the experiments, the correction is adjusted according to the silicon thickness. The measurement lens is a long working distance objective with a NA of 0.7 (Mitutoyo, NIR). The infrared imager is an InGaAs array (Raptor Photonics) with a linear response to the laser energy verified by calibration experiments. The obtained image at each position is formed on an average of 4 applied pulses. The measurement lens is focused at the rear surface of the sample to ensure that there is only air between the measurement lens and the imaged plane, so that no excited material will perturb the imaging process. Absolute fluence mapping is carefully calibrated based on integrated energy measurements and focal spot analyses in air. Details are discussed in Supplementary Note 1.

### 4.3. Sample Preparation

The silicon samples are microelectronic-grade high-resistivity crystals (Siltronix, orientation (100) < 0.5, resistivity 200 − 600 *Ω* cm), cleaved into pieces using a diamond tipped scriber. The thickness is 1 mm. The samples are double-side polished to avoid surface scattering for the incoming pulse and back-surface imaging.

## Data Availability

All relevant data are included in the paper and supplementary information files.

## Supplementary Materials

Supplementary 1 Supplementary Note 1: descriptions of the experimental setups and methods. Supplementary Note 2: measurement of the temporal shape of the ultrafast pulse trains. Supplementary Note 3: measured intensity distributions for more than 4 pulses in the ultrafast pulse trains. Supplementary Note 4: measurement of surface modification thresholds. Supplementary Note 5: dissymmetric modifications induced by linearly polarized single-pulse irradiations inside Si. Supplementary Note 6: calculation of point-spread-functions as function of focusing depth inside Si (linear propagation).

Supplementary 2 Video 1: demonstration of multilayer modifications at different positions.

Supplementary 3 Video 2: demonstration of multilayer modifications at the same position.
