# Supplementary material for: Ultrafast Laser Writing Deep inside Silicon with THz-Repetition-Rate Trains of Pulses
Source: Research (Wash D C). 2020 May 14;2020:8149764. doi: 10.34133/2020/8149764 (PMC7245002; doi:10.34133/2020/8149764)
Supplement: Supplementary 1 — Supplementary Note 1: descriptions of the experimental setups and methods. Supplementary Note 2: measurement of the temporal shape of the ultrafast pulse trains. Supplementary Note 3: measured intensity distributions for more than 4 pulses in the ultrafast pulse trains. Supplementary Note 4: measurement of surface modification thresholds. Supplementary Note 5: dissymmetric modifications induced by linearly polarized single-pulse irradiations inside Si. Supplementary Note 6: calculation of point-spread-functions as function of focusing depth inside Si (linear propagation). [file 8149764.f1.pdf]

Supplementary Notes

Ultrafast laser writing deep inside silicon with THz-repetition-rate trains of pulses

Andong Wang, Amlan Das, David Grojo
Aix-Marseille Univ., CNRS, LP3 UMR 7341, 13009 Marseille, France.

Outline

Supplementary Note 1: Descriptions of the experimental setups and methods

Supplementary Note 2: Measurement of the temporal shape of the ultrafast pulse trains

Supplementary Note 3: Measured intensity distributions for more than 4 pulses in the ultrafast pulse trains

Supplementary Note 4: Measurement of surface modification thresholds

Supplementary Note 5: Dissymmetric modifications induced by linearly polarized single pulse irradiations inside Si

Supplementary Note 6: Calculation of point-spread-functions as function of focusing depth inside Si (linear propagation)

Supplementary Note 1: Descriptions of the experimental setups and methods

Laser setup. The laser source is a femtosecond laser system (PHAROS, Light Conversion) with a central wavelength of 1028 ±5 nm and a pulse duration of ~170 fs. An optical parametric amplifier (ORPHEUS, Light Conversion) is used for wavelength conversion to 1550 nm. The energy is controlled by a combination of half wave plate and broadband polarized beam splitter (PBS). As shown in figure S1, the laser beam propagates into a home-built stretcher consisting of two gratings (Thorlabs) introducing negative group velocity dispersion in the laser pulses. The incident angle is 10° away from the Littrow angle. One grating and the retroreflector are mounted on a motorized stage so that the pulse duration of the laser pulses is simply tuned by changing the distance between the gratings (calibration not shown). Irradiation with a laser beam at the original pulse duration is also possible by bypassing the stretcher with an insertion mirror (inserting M1 and removing M2). Then, the laser propagates through a stack of birefringent crystals of different thicknesses, so that high-repletion-rate trains of pulses can be generated. The temporal profile of the pulse trains can be measured by a home built auto-correlator setup. A long delay line on this setup allows a measuring range of 160 ps with a high resolution. The beam size is expanded by two lenses (a concave lens of 75 mm focal length and a convex lens of 400 mm focal length) to overfill the entrance pupil of the micro-objectives used for interaction studies. The silicon samples are microelectronics grade high-resistivity crystals (Siltronix, orientation (100)<0.5°, resistivity 200-600 Ω.cm), cleaved into pieces by using a diamond scriber. The thickness is 1 mm. The samples are double-side polished to avoid scattering form surfaces. The silicon substrate is controlled by a precision three-dimensional stage (NEWPORT, M-VP-25XA-XYZL). During irradiation experiments, the motion of the sample and the emission of the laser pulses are computer-coordinated.

Burst generation using crystals. When a linearly polarized laser pulse propagates through a uniaxial birefringent crystal (a-cut, optical axis of the crystal perpendicular to propagation axis), the pulse temporally splits into two components orthogonally polarized along the ordinary and extraordinary axis. The amplitude ratio of the output pulses depends on the angle between the incoming polarization and the optical axis. The time delay (Δt) between the two components is directly defined by:

$$\Delta t = \frac{d \cdot (n_e - n_o)}{c}, \qquad (SE1)$$

where d is the thickness of the crystals, n_o and n_e is the ordinary and extraordinary refractive index of the crystal, and c is the speed of light. The time delay can be pre-set precisely by the thickness of the crystal. The relative amplitude of the two components can be continuously adjusted by rotating the incoming linear polarization. When the angle between the polarization and ordinary axis is 45 degrees, equal-amplitude cross-polarized pulses are generated. For a cascade of pulse splitting and the generation of a succession of pulses in a train, an arrangement of several crystals with gradually changing thicknesses is used [1, 2]. By using this approach, we engineered a stack of YVO_4 birefringent crystals with different thicknesses to generate high repetition rate pulse trains (as shown in figure 2 of the main manuscript) from single femtosecond pulses delivered by the source that we described above. Briefly, the thicknesses of the crystals are 0.25 mm, 0.5 mm, 1 mm, 2 mm, 4 mm, and 8 mm, respectively. The thickness precision is ± 0.05 mm and all surfaces of the crystals are anti-reflection coated for the 1550-nm wavelength at which the laser experiments are performed. The birefringence of YVO4 $\Delta n = n_e - n_o$ is 0.214 at 1550 nm corresponding to splitting delays of 0.18 ps, 0.36 ps, 0.71 ps, 1.43 ps, 2.85 ps and 5.71 ps depending on the crystals. The number of pulses in the trains can be simply tuned by changing the number of crystals or by inhibiting the splitting effect of a crystal by precise alignment of the input pulse polarization with a birefringence direction. By using n crystals, pulse trains of up to 2^n pulses can be created.

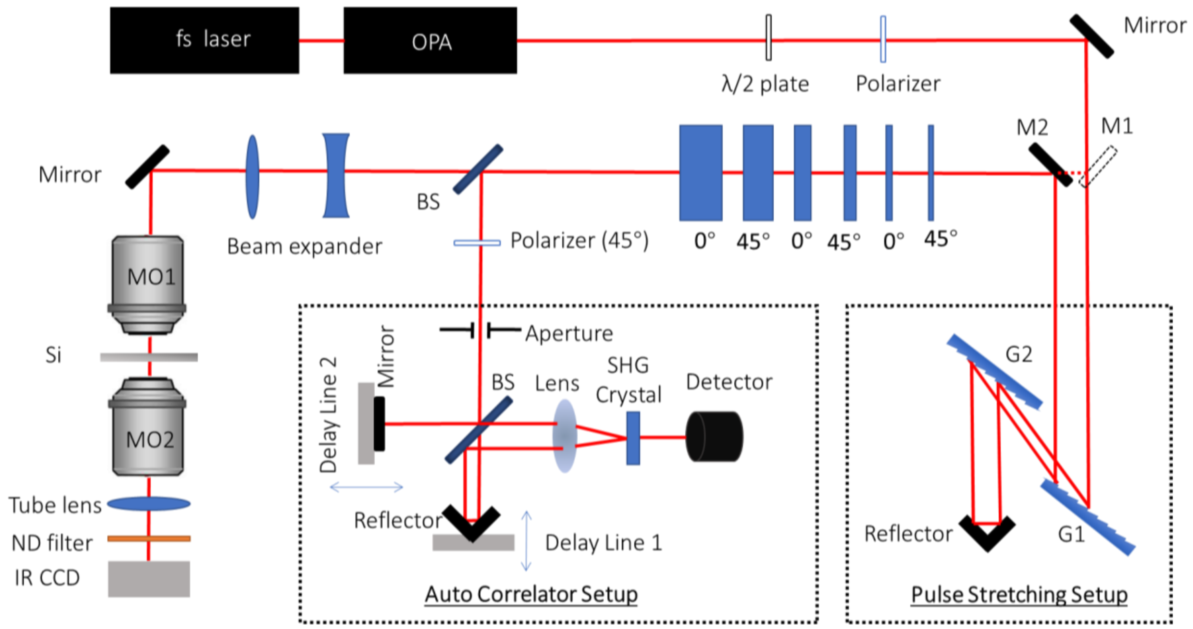

Figure S1. Simplified schematic of the experimental arrangements including the laser source, the stretcher setup, the characterization systems (autocorrelator and focus imager) and the irradiation setup. Abbreviations: OPA, Optical Parametric Amplifier; M, Mirror; G, Grating; BS, Beam Splitter; SHG, Second Harmonic Generation.

3D reconstruction of delivered and absorbed energy density distributions. The objectives used to focus the laser pulses are from Olympus LCPLN-infrared equipped with adjustable collars for spherical aberration correction due to silicon. For all the experiments, the correction is set according to the silicon thickness. The beam focus analysis is conducted using the on-axis imaging system as shown in Fig. S1 (bottom left) behind the sample. A long working distance with NA higher than that for focusing (NA = 0.7 Mitutoyo near-infrared series) is used to image the focal spot at the exit surface (back) of the sample onto an InGaAs camera (Raptor, OWL SWIR 640). The focusing objective is mounted on a motorized stage, which is indexed at 0.5 µm corresponding to ≅ 1.75 µm between images due to refractive index mismatch at Air/Si interface (z-scan procedure). Because there is no material after the imaged plane and the intensities of the measured beams are low enough to avoid any nonlinear propagation effects in air, we are dealing only with real space images. Accordingly, we directly reconstruct from the collected stack of images the full 3D fluence distributions in silicon. For all acquisitions, the laser repetition rate is 1 kHz and the array exposure time is 4 ms, so that each profile relies on an average over four laser pulses. The spatial resolution of this diagnostic is obviously subject to the diffraction limit of our NA= 0.7 observing objective. For these reasons, pump focusing with the NA= 0.85 objective is not investigated by this method. By comparing the measured distributions with simulations, we estimate the resolution and dynamic of the measurements are about 1 µm and 19 dB, respectively. A calibration procedure consisting in a focus analysis at low intensity in air and integrated energy measurement is performed. Based on the linear response of the detector at 1550 nm, the ratio of energy and pixel value can be defined as: $C=E/\sum P(x_i,y_i)$, where E is the pulse energy measured with a power meter, and $P(x_i,y_i)$ is the pixel value at a particular position. The fluence at each pixel can be then calculated based on the pixel value (accounting for the magnification) and surface of the pixel: $F(x_i,y_i)=C\times P(x_i,y_i)/S_{pixel}$. After the calibration, the intensity distribution at different position can be measured (one example of z=0 µm is shown in Fig. S2a) to reconstruct the 3D fluence distribution (see Fig. S2b) and cross-section along the propagation axis (see Fig. S2c). The

energy losses with propagation can be easily calculated by integrating the pixel values at each *z* plane. However, one has to note that local absorption (spatially resolved absorption) is hard to be obtained because the fluence changes at different positions are coupled with complex nonlinear propagation. For quantitative comparisons of the absorption densities, we estimate the peak absorbed energy density at each *Z* position by dividing the energy difference δE between two consecutive images to the local beam area (at FWHM) $S(Z)$.

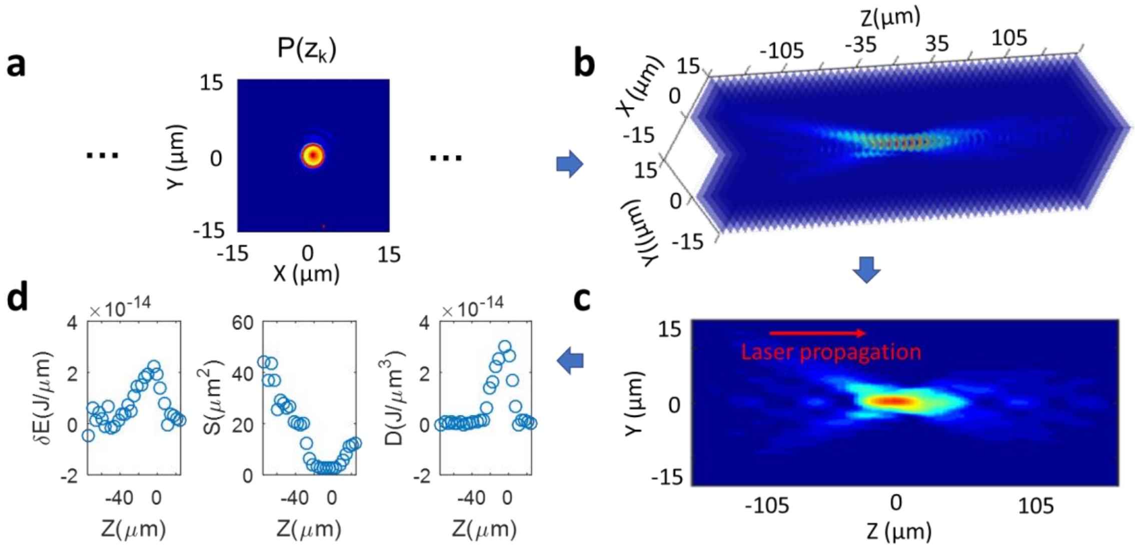

Figure S2. Example of procedure for reconstruction of 3D fluence distributions and estimation of the absorbed energy densities inside silicon. (a) Intensity distribution measured at Z=0 µm for a beam focused with NA=0.45 (geometrical focus); (b) 3D fluence reconstruction based on multiple measurements at different Z positions; (c) Cross section along YZ plane; (d) Calculation of average absorption density (D) based on energy difference (δE) and beam area (S) at adjacent measurements (Z-scan).

Supplementary Note 2: Measurement of the temporal shape of the ultrafast pulse trains

To verify the effective generation of pulse trains using the crystals, characterizations have been systematically done using the long-scan auto-correlator. For an incident laser temporal envelop E(t), the auto-correlation signal intensity $A(t)$ is:

$$A(t) \sim \int E(t-\tau)\cdot E(t)\mathrm{d}\tau, \qquad (E\text{-}S2)$$

where τ is the varying delay between the two pulses in the autocorrelation. We have simulated the auto-correlation signal intensity by using this simple equation for comparisons with measurements under various crystal configurations.

Figure S2 shows the results for trains of two pulses created by crystals of different thicknesses. All the axes of the crystals are set at 45° with respect to the polarization of incident pulse. For the measurement, the generated two-pulse trains first propagate trough a polarizer (polarization projection) for proper operation of the auto-correlator. There are two important features which can be extracted from the auto-correlation signal. First is the delay between peaks which equals the delay between the measured pulses. Second is the relative amplitude of the peaks which reveals the intensity ratio of the measured pulses. As shown in the figure S2, we have a good agreement with the theoretical delays. The intensity ratio between the main lobes and side lobes are approximately 2:1 which corresponds well with two pulses of identical intensity. We obtain an excellent agreement with the simulation results and only a noticeable deviation for the 0.25 mm crystal. With this thickness, the delay between two pulses is around 170 fs. This is approximately equal to the FWHM duration of a single pulse. Thus, interference between the two pulses during the measurement (same polarization required for the pulses) are inevitable and likely cause the observed modulation of the auto-correlation envelop.

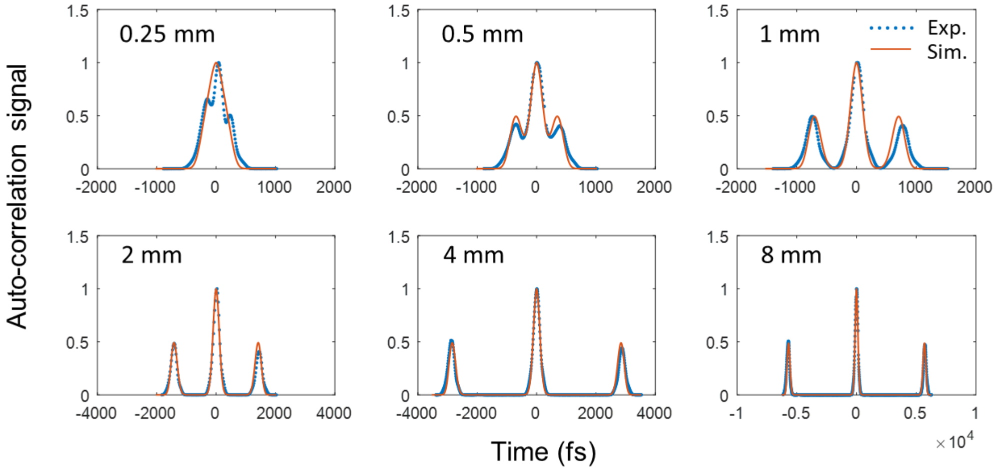

Figure S3. Comparison between experimental results (blue dots) and simulation results (solid red line) for the auto-correlation signals using crystals of different thicknesses. The thicknesses are mentioned on the upper left corner of each graph.

The auto-correlation signals for different numbers of crystals are also measured to verify the effective generation of pulse trains so-called *bursts*. These are shown in Figure S3. The delays and intensity ratios of the experimental results fits the simulations well. This evidences that we generate bursts with controlled characteristics. It is again worth noting that for the 6-crystals case, the experimental signal exhibits important fluctuations in comparison to the simulation. This is also directly attributed to the interference between adjacent pulses.

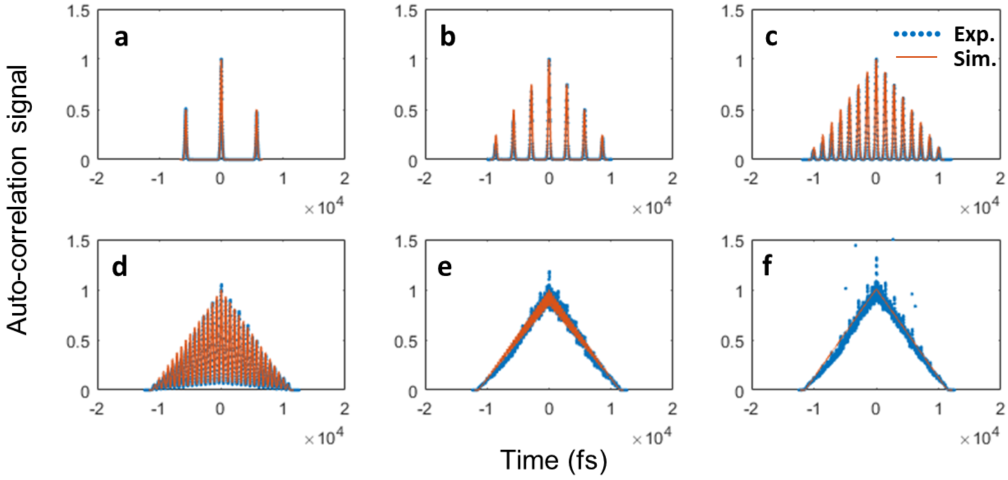

Figure S4. Comparison between measurements (blue dots) and simulation results (marked by solid red line) for different numbers of crystals. For figure (a) to (f), from one graph to the next one, one more crystal is added into the beam path (*N* from 1 to 6, respectively). The thicknesses of the crystals progressively added are 8 mm, 4 mm, 2 mm, 1 mm, 0.5 mm and 0.25 mm, respectively.

Supplementary Note 3: Measured intensity distributions for more than 4 pulses in the ultrafast pulse trains

To illustrate the general features of energy delivery by pulse trains, the measured fluence distributions that are delivered inside silicon with a double-pulse irradiation are presented in the main manuscript. The distributions measured with higher number of pulses are shown in figure S5. Considering a pulse train of 2^n pulses, the delivered fluence by the first half (from 1 to 2^{n-1} pulse) of the train is given by the pulse train of 2^{n-1} pulses at half the energy. Therefore, the contribution of the second half (pulse number from $2^{n-1}+1$ to 2^n) of the pulse trains is obtained by subtracting this first half contribution from the total fluence distribution measured with the pulse train of 2^n pulses. The results are shown in figure S6. The obtained results may reflect the level of screening effect created by the first half of the pulse trains. From the obtained results, we can obviously see the higher transmission of the second half of the trains when we increase the number of pulses in the trains. While this does not allow to access the absorbed energy densities that are directly relevant for material modification, this observation however confirm a more efficient delivery of laser energy near the focal region with the burst mode of irradiation.

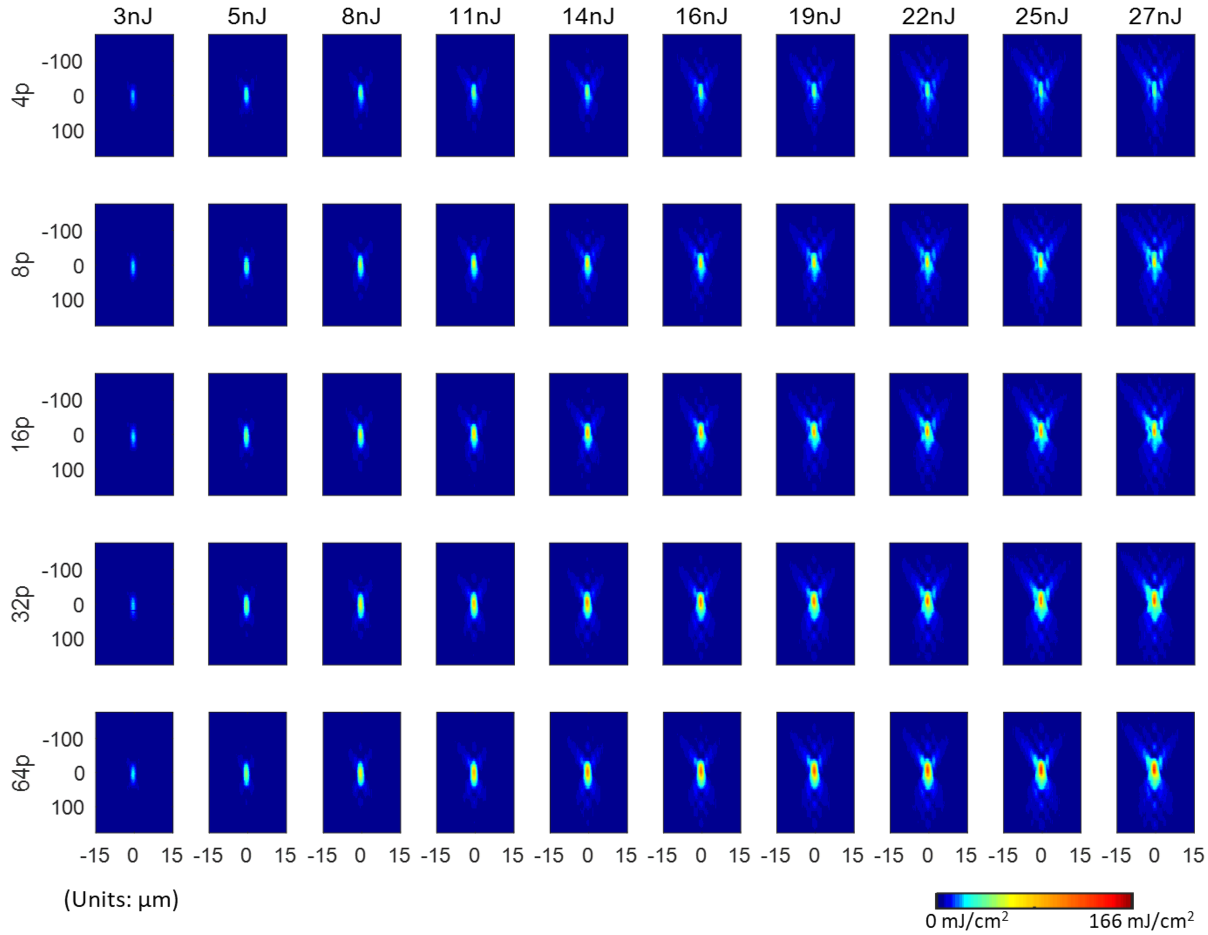

Figure S5. Delivered fluence distributions for pulse trains at different energies. To complement the double-pulse case presented in the manuscript, this shows trains with number of pulses from 4 to 64.

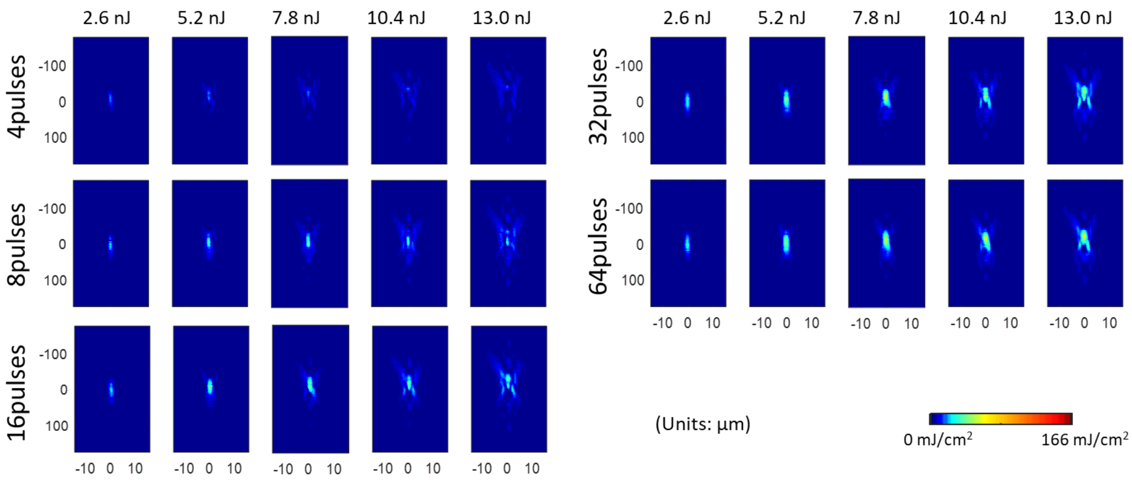

Figure S6. Contribution of the second half (pulse number from $2^{n-1}+1$ to 2^n) of the pulse trains (2^n pulses in total) at different energy levels and number of pulses in the trains. To complement the double-pulse case presented in the manuscript, this shows trains with number of pulses from 4 to 64.

Supplementary Note 4: Measurement of surface modification thresholds

For evaluation of the conditions applied to Si, the measured fluence distributions which are delivered to the bulk of Si are compared to the modification thresholds measurable with the same beam focused at the surface of the same sample. For determination of the fluence threshold for surface modification, a lens with a focusing length of 50 mm is used so that the long Rayleigh range is favorable for sample positioning near focus. We apply a single shot methodology with an incident pulse energy fixed at 2.75 µJ for all the measurements. The beam profile is first measured by using an imaging system of 50×magnification. The images of the focusing spot (inset) and its cross section are shown in figure S7a. The same focused beam is then used to modify the surface. Using different bursts for irradiation, the damaged results observed by optical microscopy are shown in figure S7b. A relatively brighter ring can be seen around the damaged spots, which can be directly attributed to the amorphization of crystalline silicon and its higher reflectivity [3]. In this measurement, the size of the circle was used to provide directly a rough estimation of the modification thresholds. By looking at the correspondence of the size of the circles to the measured fluence distribution, the threshold fluence is obtained. The results are shown in Table S1. This shows how the fluence threshold for modification increases with the apparent increase of the irradiation duration with pulse trains.

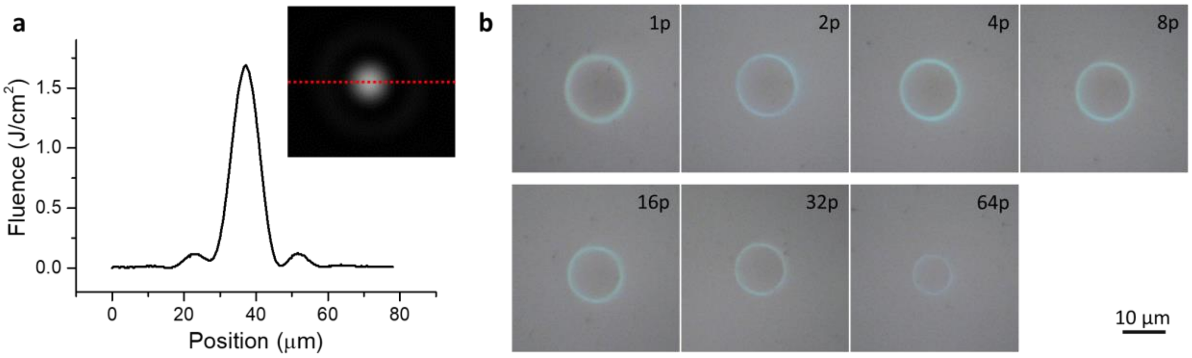

Figure S7. Laser induced damage threshold measurement. (a) Images of the focusing spot (inset) and its cross section (b) Modification results measured by optical microscopy. All the images share the same scale bar at the bottom right corner.

Pulse number	Area of measured modifications (μm^2)	Radius of measured modifications (µm)	Fluence threshold for modification (J/cm^2)
1	148	6.9	0.22
2	137	6.6	0.27
4	125	6.3	0.33
8	116	6.1	0.37
16	102	5.7	0.46
32	86	5.2	0.57
64	49	4.0	0.94

Table S1. Laser induced damage threshold results of different pulse numbers.

Supplementary Note 5: Dissymmetric modifications induced by linearly polarized single pulse irradiations inside Si

To verify that the dissymmetric structures obtained inside silicon must be attributed to the material response to the laser field, we have confirmed the symmetricity of the incident beam profile by comparison with surface damage experiments. Briefly, we have performed several experiments consisting in repeated modifications at different energies while performing Z-scan from surface to the bulk without any change on the other laser parameters. The modification results are observed by an infrared microscopy as shown in figure S8 where the horizontal axis shows results for different focusing planes and the vertical axis for different energies. In figure S8a, the microscopy imaging system is focused on the surface structures, while in figure S7b it is focused on the bulk modifications. The result shows unambiguously that the same beam creates symmetric features on the surface and dissymmetric structures in bulk silicon. This observation is very similar to another study concentrating on double-pulse femtosecond laser irradiation by Mori et al [4]. While the temporal regime is different here, it is interesting to note the use of similar focusing conditions (NA=0.85) and none of other studies in the literature report any dissymmetry.

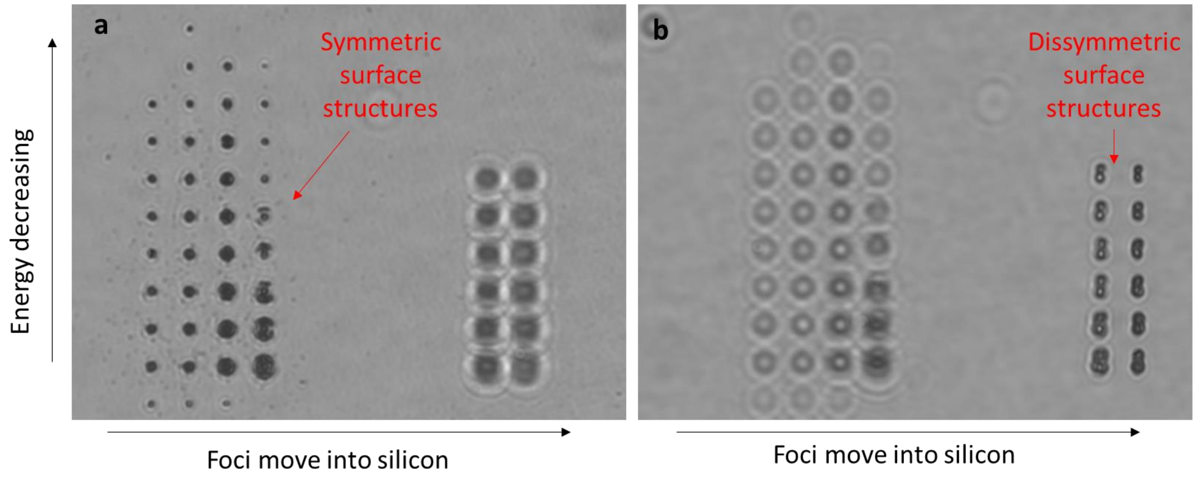

Figure S8. Comparison between surface (a) and bulk (b) modifications induced by single 6.7-ps laser pulses focused with a numerical aperture of 0.85.

Supplementary Note 6: Calculation of point-spread-functions as function of focusing depth inside Si (linear propagation)

The focal intensity distribution accounting for spherical aberration was simulated under assumption of purely linear propagation situation with no plasma generation, Kerr effect or other nonlinear effects. The simulation use an open-source software "PSFLab" [5] based the exact vectorial theory. As shown in figure S9, the simulations are repeated for the focusing conditions of our experiment (NA=0.85, correction for 300 µm thickness of Si) and changing the focusing depth. The intensity distributions (Fig. S9a) show the changes due to spherical aberration with deviation from the ideal focusing plane (here 0 µm) for correction. In figure S9b, the peak fluence extracted from the simulated distributions is shown for different focusing depth around the best focus for correction. We note that a 6% decrease of the peak fluence is expected for ±21µm deviation from the best focus.

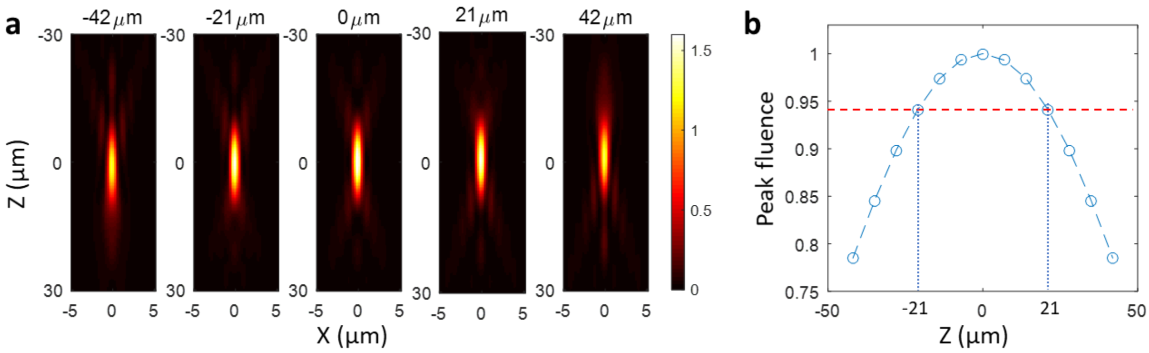

Figure S9. Effect of spherical aberration on local fluence distributions near focus inside Si. (a) Simulated intensity distributions near focus (NA=0.85, cross sections) for different focusing depths away from the best conditions for spherical aberration correction. The deviation distance is -42 µm, -21 µm, 0 µm, +21 µm, and +42 µm, respectively. (b) Peak fluence extracted from the simulated distributions and reported as a function of the deviation distance Z.

References

1. B. Dromey, M. Zepf, M. Landreman, K. O'keeffe, T. Robinson, S. M. Hooker, Generation of a train of ultrashort pulses from a compact birefringent crystal array. *Appl. Opt.* **46**, 5142–5146 (2007) DOI: 10.1364/AO.46.005142.
2. S. Zhou, D. Ouzounov, H. Li, I. Bazarov, B. Dunham, C. Sinclair, F. W. Wise, Efficient temporal shaping of ultrashort pulses with birefringent crystals. *Appl. Opt.* **46**, 8488–8492 (2007) DOI: 10.1364/AO.46.008488.
3. J. Bonse, All-optical characterization of single femtosecond laser-pulse-induced amorphization in silicon. *Appl. Phys. A*. **84**, 63–66 (2006) DOI: 10.1007/s00339-006-3583-3.
4. M. Mori, Y. Shimotsuma, T. Sei, M. Sakakura, K. Miura, H. Udono, Tailoring thermoelectric properties of nanostructured crystal silicon fabricated by infrared femtosecond laser direct writing. *Phys. Status Solidi Appl. Mater. Sci.* **212**, 715–721 (2015) DOI: 10.1002/pssa.201431777.
5. M. J. Nasse, J. C. Woehl, Realistic modeling of the illumination point spread function in confocal scanning optical microscopy. *JOSA A*. **27**, 295–302 (2010) DOI: 10.1364/JOSAA.27.000295.
